# Properties Comparison of Oxidized and Heat Moisture Treated (HMT) Starch-Based Biodegradable Films

**DOI:** 10.3390/polym15092046

**Published:** 2023-04-25

**Authors:** Yana Cahyana, Christoper Verrell, Dodo Kriswanda, Ghina Almira Aulia, Namira Azkia Yusra, Herlina Marta, Nandi Sukri, Safarov Jasur Esirgapovich, Sultanova Shakhnoza Abduvakhitovna

**Affiliations:** 1 Departement of Food Industrial Technology, Faculty of Agroindustrial Technology, Universitas Padjadjaran, Sumedang 45363, West Java, Indonesia; 2 Departement of Food Engineering, Tashkent State Technical University, Tashkent 100095, Uzbekistan

**Keywords:** film, heat moisture treatment, oxidation, starch, packaging

## Abstract

Starch-based biodegradable films have been studied for a long time. To improve starch properties and to increase film characteristics, starch is commonly modified. Amongst different types of starch modifications, oxidation and heat moisture treatment are interesting to explore. Unfortunately, review on these modifications for film application is rarely found, although these starch modifications provide interesting results regarding the starch and film properties. This paper aims to discuss the progress of research on oxidized and heat moisture-treated-starch for edible film application. In general, both HMT and oxidation modification on starch lead to an increase in film’s tensile strength and Young’s modulus, suggesting an improvement in film mechanical properties. The elongation, however, tends to decrease in oxidized starch-based film, hence more brittle film. Meanwhile, HMT tends to result in a more ductile film. The drawback of HMT film is its lower transparency, while the opposite is observed in oxidized films. The observation on WVP (water vapor permeability) of HMT starch-based film shows that the trend of WVP is not consistent. Similarly, an inconsistent trend of WVP is also found in oxidized starch films. This suggests that the WVP parameter is very sensitive to intrinsic and extrinsic factors. Starch source and its concentration in film, film thickness, RH (relative humidity) of film storage, oxidation method and its severity, plasticizer type and its concentration in film, and crystallinity value may partly play roles in determining film properties.

## 1. Introduction

Plastic packaging has become a growing environmental concern due to its extremely slow degradability characteristics, leading to soil, water, and crop contamination. Growing effort has been applied to solve the environmental problem through an application of biopolymer-based packaging, which is known to be degradable. Biopolymers commonly used as a degradable packaging matrix include carbohydrates, proteins, and lipids [1]. Amongst biopolymers, starch is the most commonly used and studied for packaging application, probably due to its abundance and cheap sources. 

As a carbohydrate polymer, starch can be found in almost all regions throughout the world. Apart from the main sources of starch, such as wheat, potatoes, and rice, other sources are considered as potential alternatives, including corn [2], arrowroot [3], cassava [4,5], sago [6], yam [7], banana [8,9,10], sweet potato [11], barley [12], millet [13], breadfruit [14], etc. Given various starch sources and the low price of raw materials, it is, therefore, unsurprising that starch is used as the most commonly biodegradable packaging matrix, including edible film.

Edible film has recently gained increasing interest from researchers and food industry [1,15]. This film is manufactured as a thin layer made from edible ingredients, covering food and serving as a barrier between food and its surroundings. Starch based-edible films have been studied [16,17,18,19], and their application as the film on cakes [20], meats [21,22], vegetables [23], and fruits [24] demonstrates an encouraging result. These findings show the prospective application of starch as edible film material.

Although starch is a promising biomaterial for edible packaging, native starch requires modification to possess these expected properties. This is because the film made from native starch often displays high sensitivity to water, brittleness, and low mechanical properties [25]. To overcome such drawbacks, starch is usually chemically, enzymatically, or physically modified [26,27,28,29,30]. Amongst starch modifications commonly applied, chemical (using oxidation) and physical (HMT) modifications are discussed in this paper. Furthermore, film properties made from oxidized and HMT starch are also explored. To the best of our knowledge, reviews on oxidized and HMT-based film characteristics are still rare. Therefore, it is pivotal to fill the gap to better comprehend the application of these types of starch modification in edible film.

## 2. Edible Film

### 2.1. Definition

Edible film can be defined as a thin layer of edible materials that is safe for consumption and used as food packaging to protect food against external factors that stimulate food deterioration, such as moisture, oxygen, light, contaminants, and many other aspects. Materials used to manufacture edible films are regarded as eco-friendly materials because they are made of edible, versatile, renewable biopolymers that can be degraded in the environment. Edible film materials are synthesized from plants, animals, and marine biota or derived from natural food-grade polymers, such as polysaccharides, proteins, or lipids [1,31,32,33,34]. 

Apart from their ecofriendly characteristics, edible films also exhibit promising abilities to protect food products, improve organoleptic properties of packaged products, and provide nutritional supplements, flavors, dyes, antimicrobial substances, and antioxidants. Furthermore, the product’s original appearance can be maintained for a longer period [35].

### 2.2. Function and Properties

Edible film serves to control O_2_, CO_2_, water vapor permeability, and to reduce the rate of destructive chemical reactions, of physical changes, and of microbial growth so that the quality of the packaged products can be maintained. Edible films are generally used as primary packaging. Therefore, the edible films are not directly exposed to factors, such as handling, storage, and transportation [36]. 

**Figure 1:**
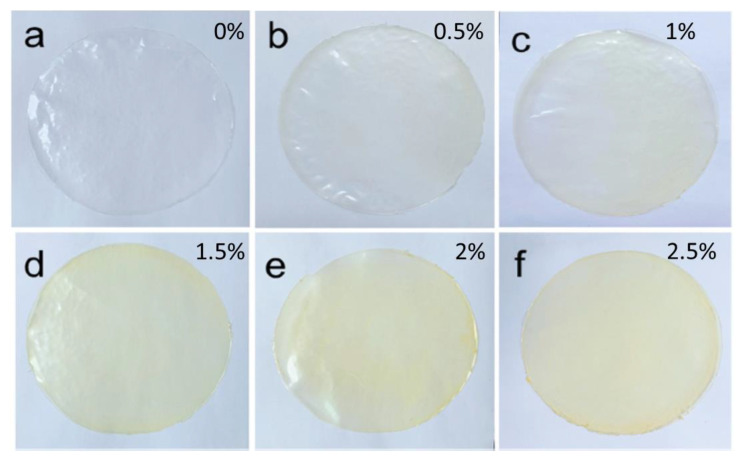
Appearance of cassava starch-based edible films (**a**–**f**) containing various concentration of cinnamon essential oil (respectively 0, 0.5, 1, 1.5, 2 and 2.5%). Reprinted with permission from [40], 2021, Elsevier.

In the manufacture of edible films, plasticizers, such as glycerol and sorbitol are added to increase the flexibility of the film [37]. Several fortifying constituents, such as hydrocolloids, lipids, and composites, can improve film functionality and protect food products from mechanical, physical, and chemical damage [38,39]. In addition, several studies have added bioactive compounds, such as essential oils and food-grade bacteriocin compounds, as antioxidants and antimicrobial agents. The application of cinnamon essential oil and the visual appearance of cassava starch-based edible film with cinnamon essential oil addition is presented in Figure 1 [40]. The addition of cinnamon essential oil into the film matrix improves film properties, such as the thermal and crystal structure stability of film, barrier properties against oxygen, and ultraviolet characteristics [40]. 

Edible films have been widely used and applied to food, especially highly perishable products, such as horticulture products. In principle, edible film packaging protects food products during the supply chain, including distribution, storage, and marketing [38].

Edible films can extend the shelf life of food products by inhibiting moisture migration and the loss of volatile compounds, reducing respiration rates, and delaying changes in texture properties. In addition, edible films are excellent coatings or membranes for fats and oils [31,32,35,38].

### 2.3. Edible Film Preparation

Edible film preparation includes the dry method and the wet method. The dry method is carried out by processing biopolymers, such as proteins and polysaccharides, at low moisture content in such a way that the film is formed due to their thermoplastic properties. This method comprises extrusion, injection, and compression molding [41,42]. The injection method and compression molding are sometimes applied in combination with the extrusion method to obtain the final thermoforming process [43]. Film properties obtained by the extrusion method are dependent on screw speed, temperature, feeding rate, and moisture content. By contrast, those obtained by the compression method are governed by temperature, pressure, and time [41,44,45]. Pre-injection and molding temperature, as well as injection pressure, affect film properties prepared by injection molding [46].

The wet method is also known as the casting method. In this method, film is formed by solubilizing or dispersing biopolymers in suitable solvents, such as water or alcohol. The heating-while-mixing process is applied to solubilize and to homogenize the dispersion. The blended mixtures are then cast onto flat, leveled, non-stick plates, followed by a drying process at ambient temperature [47]. In this method, air bubbles are often entrapped in the film matrix, creating defects in the structural integrity of films. Vacuum degassing prior to film solution casting is necessary to remove the air bubbles [48,49]. 

Another emerging method, which is deemed as an effective approach to form films, is the electrospinning technique. This technique results in a fibrous film with interesting properties [50]. Electrospun fibrous starch film is prepared by solubilizing starch into a suitable solvent while mixing at a proper temperature. The solution loaded into a syringe is subject to electrical charges through the syringe needle. The solution is pumped by a syringe pump toward an electric field. The solution then forms solidified fibers, which are collected by a collector [51].

## 3. Starch as a Material for Edible Film

### 3.1. Native Starch and Its Drawbacks for Edible Film Application

Starch is one of the few renewable resources that can form films and meets all the key criteria, including ease of availability, high extraction yield, nutritional value, affordability, biodegradability, biocompatibility, and edibility, thus possessing functional qualities. It is, therefore, a potential substance for edible coatings and films [19,52,53]. In addition, such films are non-toxic, tasteless, colorless, odorless, and semipermeable to lipid and flavor components, carbon dioxide, moisture, and oxygen [54]. 

However, native starch has various undesired shortcomings due to its semi crystalline (20–45%) structure, including its hydrophilic nature, poor solubility, poor mechanical qualities, uncontrolled paste consistency, and low freeze–thaw durability during film development [55]. Many modification methods, such as physical, chemical, enzymatic modifications, and additive additions, can be used to fix these defects and enhance the features of the starch film. These would change the molecular structure of starch to improve its characteristics [54]. Amongst various starch modifications, oxidation and HMT are discussed in this paper, along with their application in edible films. 

### 3.2. Starch Modification (Oxidation and Heat Moisture Treatment)

#### 3.2.1. Oxidation

**Figure 2:**
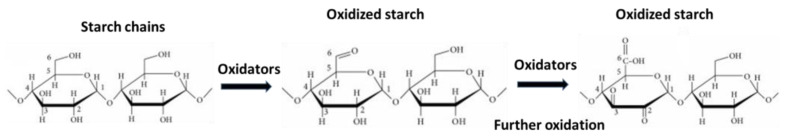
Oxidation of starch hydroxyl groups to form carbonyl and carboxyl groups.

Oxidation is classified as one of the chemical modification methods widely used to modify starch. Oxidation modification involves the oxidation reaction of primary or secondary hydroxyl groups, which results in carbonyl or carboxyl groups [56]. The schematic of the starch oxidation reaction is presented in Figure 2. The oxidation reaction may be affected by many factors, including the oxidant type, starch source, and environmental conditions. Furthermore, the oxidation reaction may result in loosening intermolecular bonds or depolymerizing the polymer chains. Oxidation modification may be performed by different oxidant types, such as oxygen, hydrogen peroxides (H_2_O_2_), sodium hypochlorite (NaClO), sodium periodate (NaIO_4_), nitrogen compounds (HNO_3_, N2O_4_), organic oxidants, as well as metal compounds (CrO_3_). However, among the wide choice of oxidizing agents, sodium hypochlorite (NaOCl) and ozone (O_3_) are some of the most used modifying agents [57,58,59]. 

**Figure 3:**
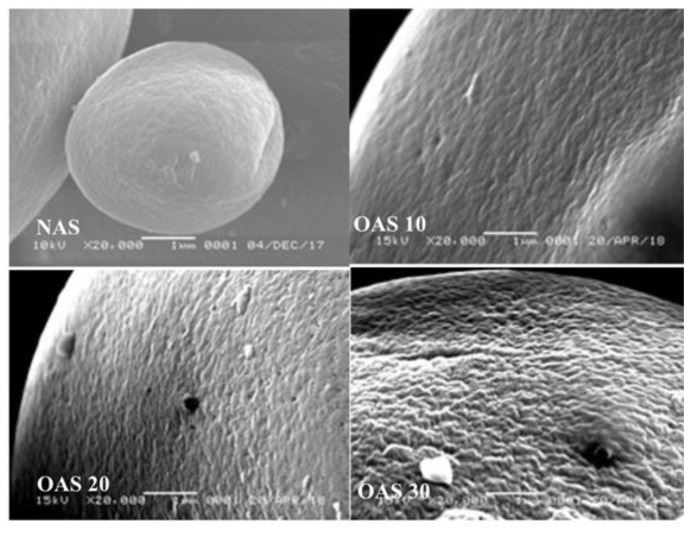
Granular morphology of native (NAS) and ozonated starch (OAS 10, OAS 20, and OAS 30). Reprinted with permission from [3], 2020, Wiley-VCH Verlag.

Starch oxidized by ozone can be modified in dry form or in the form of aqueous suspension. In terms of processing practicality, dry starch ozonation possesses several advantages over its wet counterpart. Dry starch ozonation is not affected by pH of milieu during ozonation. Furthermore, it does not require a re-drying step following ozonation, thereby using less energy and having a shorter time of oxidation. Dry starch ozonation, also known as gaseous ozonation, has been proved to alter granular morphology of starch (Figure 3). Carbonyl content, crystallinity, functional properties, and thermal properties, such as onset (To), peak (Tp), and conclusion (Tc) transition points, as well as the difference between Tc and To (ΔT) and enthalpy change (ΔH), are also altered following ozonation in the dry process. The authors observed the increase in To and ΔH of ozonated starch, suggesting that ozonation leads to a formation of stronger and stable crystallites [3].

Commonly, starch modified with oxidation possesses low viscosity, high thermal stability, low retrogradation, high paste clarity, good film forming, and high binding properties. Besides being widely used as a food additive in the food industry because of its specific properties, oxidized starch also is commonly applied in other industries, such as in textiles or paper fabrication [56]. When applied in food preparations containing lipids, ozonated starch displays higher starch–lipid complex value. This interaction changes the swelling volume, gel strength, and freeze–thaw stability of dough [58].

#### 3.2.2. Heat Moisture Treatment (HMT)

Heat moisture treatment (HMT), a physical (hydrothermal) method, is used to modify the starch structure and functional properties without damaging the granular structure of the starch. The process is strongly influenced by several factors, including the level of humidity, type of starch, heating time, and temperature [60]. In principle, starch with low water content (<30%) is heated above its glass transition temperature, but below its gelatinization temperature, for a certain period [9,61].

HMT modification leads to the disruption of starch crystalline structure and dissociation of double helix structures. At the end of the process, rearrangement of the disrupted crystalline structure occurs. These changes strengthen the interaction between amylose and amylopectin branching [62,63]. As a result, HMT starch exhibits lower freeze–thaw stability, lower swelling volume and water absorption capacity, higher pasting temperature, and higher pasting stability. The changes in morphology, swelling capacity, crystallinity, gelatinization, retrogradation, and digestibility are also observed in other studies on HMT starch [64,65,66,67]. 

The increase in the physicochemical properties of HMT starch has broad potential for industrial and laboratory applications that require starch with high gelatinization temperature [63]. When applied in food products rich in lipids, HMT starch shows a higher ability to form a complex with lipids than native starch, leading one to alter paste or dough characteristics. The breakdown viscosity of HMT starch, for instance, decreases in the presence of lipids, suggesting that the paste or dough is more stable to shear and heat treatment [68]. 

## 4. Starch Modification Effect on Edible Film Properties

Film properties made from oxidized and HMT-starch, such as mechanical properties, permeability to water vapor and gas, film solubility, transparency, and mechanical properties, are presented in Table 1. 

### 4.1. Gas (O_2_) and Water Vapor Permeability (WVP)

Adequate food packing depends on the packaging material’s capacity to prevent or reduce gas or moisture transfer between the food and the environment. The best barrier characteristics of edible films against WVP and O_2_ gas are given by their lowest permeability [81]. The value of WVP is a function of water solubility and diffusivity through the film. For starch-based film, other factors, such as plasticizer content, water activity gradient, film thickness, and temperature, should be taken into account when estimating the permeability value [82,83].

Films made from HMT rice starch exhibit higher WVP than native starch films [70]. A similar finding was also found for potato and amaranth starch films [62,73]. Interestingly, a decrease in WVP is observed in some other studies on sweet potato and green plantain banana starch films [74,79]. 

It is not clear whether the starch source affects the WVP of HMT film. However, film thickness has been found to affect the WVP of film [84,85]. Moreover, the presence of micro-cracks, uneven surfaces, and pores on the film’s surface may increase WVP [86]. Further study on HMT starch-based edible film is necessary, taking into account factors, such as thickness and surface characteristics. The presence of pores and micro-cracks may be identified using microscopic methods to ensure that the analysis of WVP is well carried out. 

Ozonized cassava starch-based film shows an increase in oxygen permeability and WVP [69]. The increase in WVP is also found in another study using banana starch and barley oxidized with sodium hypochlorite [75,76,77]. However, the opposite results of a film’s WVP have been shown in other studies using potato, amaranth, and sorghum starch oxidized with sodium hypochlorite [62,71,72,73,80]. Oxidized potato starch-based film possesses lower WVP than native potato starch film [62]. This finding is in agreement with other studies on the oxidized potato starch-based films [71,72]. The consistently lower WVP of the films made from oxidized potato starch suggests that the starch source might affect the resulting WVP. Furthermore, studies on film permeability to O_2_ are rarely found. A study on oxidized cassava starch-based film shows an increased permeability of film to O_2_ [69]. 

Oxidation of starch allows the formation of carboxyl and carbonyl groups. Initially, hydroxyl groups of starch are oxidized to carbonyl groups. Further oxidation forms carboxyl groups, along with an increase in the hydrophilicity of oxidized starch. Oxidation level is estimated according to the number of carboxyl and carbonyl groups of the oxidized starch [87,88].

The discrepancy in the WVP trend of oxidized films may be explained by several factors. The first one is the presence of cracks in the film. Cross-section observation on ozonized cassava starch-based film shows the presence of cracks, despite its compact structure and smooth surfaces [69]. These cracks may facilitate the permeation of water vapor and gas. The second one is that different levels of oxidation, indicated by the number of carboxyl and carbonyl groups, lead to different films with different hydrophilicity or hydrophobicity. This may also increase free space between glucan chains, facilitating the interaction with plasticizer to form more compact films. The distance between macromolecules caused by the molecular charge of carboxyl groups increases the network permeability to oxygen or water vapor [80]. Therefore, the different WVP of oxidized starch based-films compared to their native starch films might partly result from the different degree of oxidation, leading to different hydrophilicity levels and/or molecular free space in the films.

### 4.2. Contact Angle

**Figure 4:**
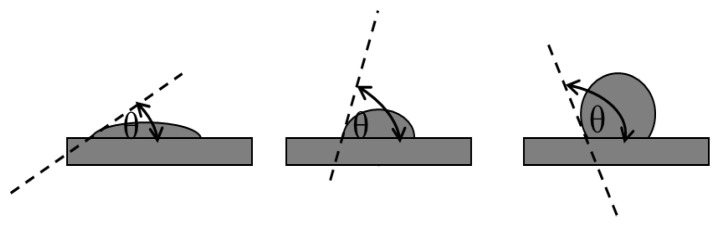
Illustration of contact angle between films and a liquid drop.

Controlling the wettability parameters, such as contact angle, surface energy, surface tension, and forces of adhesion and cohesion, is crucial for the success of edible films [36]. Contact angle measurements provide a quantitative method for describing starch film surface characteristics [89]. A contact angle is the angle formed between the perimeter of a liquid drop and the surface on which the liquid drop rests. Typically, the contact angle is applied to determine surface hydrophilicity or hydrophobicity. Hydrophobic surfaces are defined by the higher contact angle, usually ≥90°, while the contact angle of hydrophilic surfaces is usually ≤90°. Time-lapse photos taken from a contact angle goniometer demonstrate how the morphology of the liquid droplets that are deposited on the film surface varies over time. At equilibrium, the contact angle is then calculated [90]. An illustration of the contact angle between films and a water drop is presented in Figure 4. 

A study on ozonized cassava starch-based film shows that the contact angle of film decreases with the increase in ozonation time, suggesting that the film is more hydrophilic with ozonation. Interestingly, when analyzing its solubility, the film is less soluble with ozonation [69]. Given that ozonation leads to an increase in the carbonyl and carboxyl functional groups, the decrease in solubility and the increase in the film surface hydrophilicity indicate that the carbonyl and carboxyl groups present on the surface of the film favor bonds with the water drop on the film during the contact angle experiment, resulting in hydrophilic surface properties [69].

Film with dually modified (OSA-HMT) sago starch matrix indicates its higher initial water contact angle compared to its native starch film, suggesting that dual modification increases the hydrophobicity of the film surface. A single OSA modification of starch leads to an increase in the hydrophobicity of the film, while the contact angle of single HMT starch film failed to be measured because the surface could not hold the water drop on the surface. Therefore, the increase in the hydrophobicity of film can be attributed to the role of OSA starch. The effect of HMT starch on the surface hydrophobicity is not clear [78]. 

### 4.3. Transparency

The transparency of starch paste is essential because it is related to the visual acceptability of edible film. Oxidation of starch results in more transparent film, as observed in the study on ozonized cassava starch-based film. Better clarity, however, was observed when the starch was ozonized at longer time, suggesting the requirement of more severe oxidation [69]. A study on potato starch, oxidized by sodium hypochlorite, shows invariant transparency of film [62,72]. The difference in the transparency in film could be affected by the different charge and size distribution of starch molecules in film [69].

Film matrix made from HMT rice, potato, and amaranth starch displays lower transparency [62,70,73]. Starch tends to be darker following HMT treatment, which might be linked to the high temperature during HMT [64]. Therefore, the less transparent film results from the characteristics of darker HMT starch compared to the native one. 

### 4.4. Mechanical Strength (Modulus, Elongation, and Tensile Strength)

Mechanical strength is an important parameter, referring to the ability of film to withstand an applied load without failure or deformation. The parameters used to describe the mechanical strength are Young’s modulus, elongation, and tensile strength. Tensile strength is the maximum force (stress) that a given substance can withstand without tearing apart. Young’s modulus indicates a stiffness of material expressed as the ratio between the stress and the strain of the material. Meanwhile, elongation, which is a measure of material ductility, is expressed as the ratio between changed length and initial length of the material.

Film made of ozonized cassava possesses higher tensile strength and Young’s modulus, but less elongation [69]. Similar results are also observed on the films made of potato, amaranth, sorghum, and barley starch, which are oxidized by sodium hypochlorite [62,73,77,80]. An increase in both tensile strength and elongation is observed on the film made from oxidized green plantain banana starch [75,76]. 

Although a study on oxidized potato starch-based film found a decrease in tensile strength [71], most studies [62,69,73,75,76,77,80] reported an improvement in mechanical strength, particularly tensile strength and Young’s modulus. The enhancement in mechanical properties might be linked to the presence of carboxyl and carbonyl groups, resulting from the oxidation of hydroxyl groups. These groups provide stronger hydrogen bonds with hydroxyl groups of amylose and amylopectin polymers, leading to a more rigid film and a reduced elongation [76]. 

Film prepared from HMT starch also generally shows an increase in tensile strength, elongation, and Young’s modulus, as observed from the studies using potato, amaranth, plantain banana, and sweet potato starch [62,73,74,79]. However, a study on HMT rice starch-based film found a decrease in tensile strength, Young’s modulus, and elongation [70]. 

Broadly speaking, as far as the mechanical properties are concerned, both oxidized and HMT starch tend to result in a film with higher tensile strength and Young’s modulus. The elongation tends to decrease in oxidized starch based-film, hence resulting in more brittle film. That is, HMT starch tends to result in a more ductile film. 

### 4.5. Thermal Properties and Crystallinity 

#### 4.5.1. Crystallinity 

Crystallinity is a crucial factor in determining starch’s mechanical and thermal properties. Crystallinity is an essential property of polymers that shows the bonds between molecular chains to produce a more ordered molecular arrangement. High crystallinity properties cause increased stress and stiffness. Each polymer chain structure from different starch sources has a different crystallinity. The degree of crystallinity is directly related to the amount of amylopectin, while the amorphous phase is directly related to the amylose contained in this polymer [72]. Crystallinity is one of the critical parameters to be monitored because it considerably influences the output of the resulting film. 

Starch modification could affect not only its crystalline type, but also crystallinity level. Observation on sago and sweet potato starch, modified with HMT, shows an increase in its relative crystallinity [78,79]. A crystallinity increase in HMT banana starch was also reported [64]. The crystallinity level is believed to partly contribute to the mechanical properties of film. However, relating the degree of starch crystallinity prior to film formation with the resulting film properties is not relevant. It is worth bearing in mind that the starch of film matrix has been subject to gelatinization and then retrogradation during film preparation. As a result of this process, the degree of starch crystallinity in the film matrix is different to that in the raw starch prior to film formation. Therefore, in order to have relevant interpretation on the relation between the degree of crystallinity and film properties, it is the relative crystallinity of film, per se, which is required to be estimated.

The oxidation of sorghum starch does not change its crystalline type, nor its relative crystallinity [80]. However, although the crystalline type does not change, the oxidation using ozone to arrowroot starch leads to a decrease in crystallinity [3]. This discrepancy may be ascribed to the oxidation severity, starch source, and the presence of non-starch compounds on the starch granules. Oxidation of sorghum in this study results in 0.06% carboxyl and 0.03% carbonyl groups, following oxidation [80]. By contrast, in the arrowstarch, the carboxyl content amounts to approximately 0.5% [3]. Furthermore, a study on the oxidation efficacy of banana starch compared to its flour form shows that the non-starch content reduces effective oxidation to starch polymer [59]. 

A study on the film made from oxidized cassava starch indicates that the film crystallinity increases compared to film from native cassava starch [69]. This result confirms that, although oxidation could decrease the crystallinity of raw starch, the crystallinity value of the corresponding film could change during the preparation process and to the addition of other compounds, such as plasticizers. The increase in the crystallinity in the cassava starch-based film is concomitant with the increase in its tensile strength and Young’s modulus [69], suggesting that film crystallinity may partly play a role in film mechanical properties.

#### 4.5.2. Thermal Properties 

HMT modification of sago starch increases starch thermal stability, as indicated by the increase in the onset (To), peak (Tp), and conclusion (Tc) of gelatinization temperature and the decrease in gelatinization temperature range (ΔT) and its enthalpy [78]. This observation is in agreement with other studies on buckwheat starch, sago and arenga starch, and glutinous rice starch [91,92,93]. Meanwhile, the oxidation of starch leads to the formation of more homogenous and stronger crystallites, as observed from the increase in To, as well as enthalpy, but a decrease in ΔT of the gelatinization of sago and banana starch [3,80]. 

The parameters of gelatinization temperature measured by DSCs, such as To, Tp, and Tc, are the indicators of the degree of starch thermal stability. HMT and oxidized starch display a trend of higher thermal stability compared to their native forms. However, in the packaging matrix, not only starch affects the packaging thermal properties, but also other compounds added to the matrix, such as plasticizer. For instance, glycerol, which is commonly used as plasticizer, can decrease film thermal stability when added into film formulation [94].

## 5. Conclusions and Future Research

Modified starch (oxidation and HMT)-based edible film displays interesting features in terms of mechanical properties, permeability, transparency, and solubility. Starch modification by HMT and oxidation results in an increase in film’s tensile strength and Young’s modulus. Oxidized starch-based films tend to possess lower elongation, while the opposite holds true for HMT films. In terms of film transparency, HMT leads to less transparent films, which might be related to the high HMT temperature during starch modification. On the other hand, oxidized starch-based films exhibit higher transparency.

In terms of WVP (water vapor permeability) of HMT and oxidized films, the variations of this property are not consistent from one study to another. This property is not necessarily related to the solubility parameter and the contact angle value, as the contact angle value represents the film surface property, while the WVP is governed by many other factors, such as film structure, thickness, area, etc.

Broadly speaking, film properties are determined by factors, such as starch source, oxidation level, film thickness and crystallinity, relative humidity (RH) at mechanical properties measurement, plasticizer type used in the film, the presence of cracks or holes, etc. Oxidation levels could result in different degrees of crystallinity, leading to different film properties. 

Given the interesting result of, particularly, mechanical properties and the inconsistent results for other film properties, future research should be directed toward revealing and proving the factors affecting the film properties. Oxidation level must be taken into account. Furthermore, the film should be prepared in such a way that the thickness is similar for all tested films. As such, the film comparison properties would be more reliable. Application of both HMT and oxidized starch-based film is also interesting to further examine.

## Figures and Tables

**Table 1:** Characteristics of edible films made from ozonated and HMT-starch.

Starch Source	Modification Type and Method	Effect on Film Properties	Reference
Cassava starch	Oxidation with ozone	Increased tensile strength and Young’s modulus, but less elongation. Increased oxygen and WVP Increased film clarity at longer ozonation time. More hydrophilic surface and reduced solubility.	[69]
Rice Starch	HMT	Decreased film transparency Decreased tensile strength, elongation breaks, and Young’s modulus. Increased WVP value.	[70]
Potato Starch	HMT	Invariant solubility Lower film transparency Higher elongation, tensile strength, and Young’s modulus Higher WVP	[62]
	Oxidation by sodium hypochlorite	Lower solubility Invariant film transparency Lower elongation, but higher tensile strength and Young’s modulus Lower WVP	[62]
	Oxidation by sodium hypochlorite	Lower solubility Lower tensile strength Lower WVP	[71]
	Oxidation by sodium hypochlorite	Invariant film transparency Higher solubility Less WVP	[72]
Amaranth Starch	HMT	Increased tensile strength Increased WVP value, but decreased water solubility Increased film yellowness	[73]
	Oxidation with sodium hypochlorite	Decreased WVP and water solubility Increased tensile strength	
Green plantain banana	HMT	More cohesive matrix Increased mechanical properties (tensile strength, elongation, and modulus) Decreased WVP Invariant solubility	[74]
	Oxidation with hypochlorite	Increased tensile strength and elongation Higher WVP	[75]
	Oxidation with hypochlorite	Higher WVP Higher tensile strength and elongation	[76]
Barley	Oxidation with sodium hypochlorite	More homogenous film Increased water solubility Increased WVP Decreased elongation, increased Young’s modulus Increased tensile strength at 1.5% of hypochlorite, but not at 1 and 2%	[77]
Sago	Heat moisture treatment (HMT)	Invariant WVP Increased water solubility, WAC, and oil absorption capacity (OAC)	[78]
Sweet potato	Heat moisture treatment (HMT)	Higher tensile strength and elongation Lower WVP and solubility	[79]
Sorghum	Oxidation with chlorine	Lower WVP Higher tensile strength and Young’s modulus, but lower elongation Invariant solubility	[80]

## Data Availability

Not applicable.
